# Perspectives for Advancing Biotechnological Succinic Acid Production

**DOI:** 10.1111/1751-7915.70363

**Published:** 2026-04-28

**Authors:** Christoph Gunkel, Bastian Blombach

**Affiliations:** ^1^ Microbial Biotechnology, Campus Straubing for Biotechnology and Sustainability Technical University of Munich Straubing Germany; ^2^ SynBiofoundry@TUM, Technical University of Munich Straubing Germany

**Keywords:** industrial biotechnology, low‐pH fermentation, metabolic engineering, one carbon metabolism, succinic acid

## Abstract

Succinic acid has been considered an important molecule in the transition of chemical manufacturing from fossil‐based to sustainable and future‐proof processes. While there has been extensive research on biotechnological succinic acid production from biomass, attempts to roll out bio‐succinic acid are impeded by its high price and remaining sustainability issues. Both drawbacks are interconnected and can be traced back to the used feedstocks and a wasteful expenditure of acid and base, among others. In this opinion, we discuss biochemical principles and metabolic pathways of next‐generation carbon assimilation and low‐pH fermentations to address these drawbacks. For this reason, we chart the potential for producing succinic acid from sustainable next‐generation feedstocks based on electron, carbon and ATP balances as well as relevant thermodynamic considerations. Furthermore, we summarize key advances in low‐pH succinic acid synthesis using acid‐tolerant yeasts and assess the suitability of selected acid tolerance mechanisms for industrial application. Eventually, we aim to inspire researchers to synthesize innovative approaches to realize competitive and sustainable biotechnological succinic acid production.

## Graphical abstract

Succinic acid is an important molecule for sustainable chemical manufacturing, but its industrial production faces high costs and sustainability challenges, which are linked to feedstocks and excessive acid/base consumption. This opinion explores production routes based on next‐generation feedstocks and low‐pH fermentations to inspire novel cost‐competitive, sustainable bio‐succinic acid production approaches.

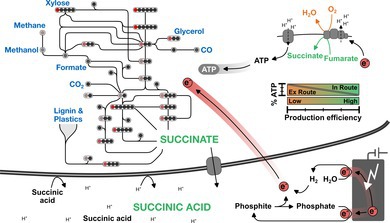

## 1 Succinic Acid—A Rocky Path Towards Sustainable Manufacturing

Anthropogenic climate change is a major threat to the global community and environment. First eco‐systems are severely (if not irreversibly) damaged, millions of people are threatened by food insecurity and climate‐associated health risks and mortalities are prevalent (IPCC 2023). To mitigate the consequences of the climate crisis, almost all countries agreed on limiting the man‐made increase in global average temperatures to below 2°C in the 2015 Paris agreement. Restricting global warming to below 2°C requires far‐reaching changes to rigorously cut greenhouse gas emissions, particularly in sectors such as buildings, agriculture, transport, industry and energy (IPCC 2023). To develop solutions to face the hunger of chemical manufacturing for fossil resources, the US Department of Energy evaluated the potential of deriving various chemicals from biomass. These assessments identified succinic acid as having high potential for production from biomass and conversion into several industrial commodity chemicals (Werpy and Petersen 2004; Bozell and Petersen 2010; Biddy et al. 2016). In the following years, various microorganisms were metabolically engineered to synthesize succinic acid from biomass at high yields and productivities (Ahn et al. 2016; Liu et al. 2022; Schmollack et al. 2024; Korka et al. 2025). In the advent of this progress, several companies—with Succinity, Reverdia, BioAmber and Myriant at the forefront—attempted to commercialize biotechnological succinic acid production (Choi et al. 2015; Ahn et al. 2016; Biddy et al. 2016). What many actors in industrial biotechnology and sustainable development hoped to be an important achievement could, however, later not keep up to the expectations. BioAmber failed for bankruptcy in 2018, Reverdia was dissolved in 2019 and its succinic acid process is now operated by Roquette, and the operation state of the Myriant and Succinity processes is unclear (Li and Mupondwa 2021).

The difficulties in rolling out bio‐succinic acid are complex, process‐specific and difficult to assess externally. Still, some studies later attempted to analyse the once running processes (Dickson et al. 2021; Li and Mupondwa 2021; Mancini et al. 2022). Two major interconnected factors were identified to have impeded the commercialization in the retrospective: Bio‐succinic acid was not cost‐competitive with petrochemically sourced equivalents and market projections heavily overestimated the demand for succinic acid. The selling price of bio‐succinic acid is, e.g., determined by company structures, financing and technology specificities as well as licensing details (Dickson et al. 2021; Li and Mupondwa 2021; Mancini et al. 2022). Regardless, pH control requirements and the feedstock (pretreatment) generally impose a relevant economic burden on biotechnological succinic acid production (Dickson et al. 2021; Mancini et al. 2022).

Most bacterial, various yeast‐based succinic acid fermentations (Table 1) (Korka et al. 2025), and putatively the Succinity and Myriant processes were operated at circumneutral pH (Ahn et al. 2016). This requires large amounts of base to stabilize the pH as succinic acid is produced, while acidification might still be necessary later for succinic acid recovery depending on the downstream process configuration (López‐Garzón and Straathof 2014; Morales et al. 2016; Dickson et al. 2021; Mancini et al. 2022). This intensive consumption of acid and base, but likely also the disposal or recovery of the accompanying salt fraction, is expensive (López‐Garzón and Straathof 2014; Dickson et al. 2021; Mancini et al. 2022). Solely the base and acid requirements were estimated to potentially account for up to 30% of the raw material costs in different processes (Mancini et al. 2022).

**TABLE 1:** Overview of selected biotechnological processes for succinic acid production.

Strain	Substrate(s) and medium	Process mode	pH	Titre | g L^−1^	Yield | g g^−1^	Productivity | g L^−1^ h^−1^	Duration	References
*Yarrowia lipolytica* Hi‐SA‐YlCA	Glucose, CO_2_ (chemically defined medium with corn steep powder)	Fed‐batch	Final pH below 3 (uncontrolled)	89.75	1.01	n.a.	n.a.	Tao et al. (2025)
*Yarrowia lipolytica* E5O1XF	Glucose (chemically defined medium with corn steep powder)	Fed‐batch	3.5 (controlled)	112.54	0.67	2.08	n.a.	Zhong et al. (2024)
*Issatchenkia orientalis* EcGS‐FF‐*gltA*	Glucose, CO_2_ (chemically defined medium with corn steep liquor)	Fed‐batch	3.0 (controlled)	101.86	0.8	0.94	108 h	Tran et al. (2025)
*Issatchenkia orientalis* g3473Δ/PaGDH‐DAK/ScSUC2	Sugarcane juice medium, CO_2_	Pilot‐scale batch	3.0 (controlled)	63.1	0.5	0.66	96 h	Tran et al. (2023)
*Vibrio natriegens* Succ1 ∆*cspR*	Glucose, CO_2_ (chemically defined medium)	High‐inoculum two‐stage batch	7.5 (controlled)	51	0.9	8.50	6 h	Schulze et al. (2024)
*Escherichia coli* JC‐211	Glucose, CO_2_ (chemically defined medium)	Two‐stage batch	7.0 (controlled)	42.48	0.91	n.a.	72 h	Chen et al. (2014)
*Corynebacterium glutamicum* CRZ21	Glucose, CO_2_ (chemically defined medium)	High‐inoculum two‐stage fed‐batch with cell recycling	7.5 (controlled)	634 g^a^	0.71	n.a.	572 h	Jojima et al. (2016)
*Mannheimia succiniciproducens* PALK (pMS3‐*mgtB*)	Glucose, glycerol, CO_2_ (chemically defined medium)	High‐inoculum two‐stage fed‐batch	6.5 (controlled)	152.23	1.30 mol mol^−1^ (glucose equivalent)	11.71	n.a.	Kim et al. (2024)
*Mannheimia succiniciproducens* PALK (pMS3‐*pelB*‐*cti*)	Glucose, glycerol, CO_2_ (chemically defined medium)	Fed‐batch	6.5 (controlled)	97.1	1.26 mol mol^−1^ (glucose equivalent)	3.01	n.a.	Ahn et al. (2018)
*Corynebacterium glutamicum* WMB2_evo_	Glucose, xylose (chemically defined medium)	Two‐phase fed‐batch	7.0 (controlled)	11.36	0.09	0.18	n.a.	Tenhaef et al. (2021)
*Corynebacterium glutamicum* BOL‐3 (pAN6‐*gap*)	Glucose, formate, CO_2_ (chemically defined medium)	High‐inoculum two‐stage fed‐batch	6.9 (controlled)	134	1.1	2.44	53 h	Litsanov et al. (2012)
*Synechococcus elongatus* PCC 7942 CR8	CO_2_ (air), photoenergy (chemically defined medium)	Re‐inoculated fed‐batch	10.0 (controlled)	8.9	n.a.	0.003–0.006	112 days	Lai et al. (2022)
*Methylomonas* sp. DH‐1 CS405	Methane, CO_2_ (chemically defined medium)	Continuous stirred tank reactor	6.85–6.95 (controlled)	0.702	n.a.	0.006	n.a.	Jo et al. (2024)
*Methylomonas* sp. DH‐1 DS‐GL	Methane, CO_2_ (chemically defined medium)	Fed‐batch	6.9 (controlled)	0.195	0.079	n.a.	n.a.	Nguyen et al. (2019)
*Methylotuvimicrobium buryatense* 5GB1S SC08	Methane, CO_2_ (chemically defined medium)	Fed‐batch	n.a.	0.299	n.a.	0.008	36 h	Wang et al. (2024)
*Yarrowia lipolytica* Yl‐005	Methanol (complex medium)	Two‐stage batch	n.a.	0.92	0.15	n.a.	n.a.	Zhang et al. (2023)
*Vibrio natriegens* XG251	Formate (chemically defined medium)	Two‐stage batch	7.0–8.0 (controlled)	1.37	0.15	0.057	24 h	Li et al. (2025)
*Escherichia coli* BL21(DE3) S1083	Xylose, CO_2_ (complex medium)	High‐inoculum two‐stage batch	Initial pH of 7.0 (uncontrolled)	2.09	0.14	n.a.	36 h	Zhou et al. (2025)
*Cupriavidus necator* H16 SA13	Palmitic acid, CO_2_ (complex medium)	Batch	7.0 (controlled)	3.6	0.24	n.a.	48 h	Li et al. (2024)
*Phanerochaete chrysosporium*	Synthetic lignin (chemically defined medium)	Two phase batch	n.a.	0.019	n.a.	n.a.	25 days	Hong et al. (2017)

^a^ Indicated is a mass instead of a concentration.

Former industrial actors appear to have mostly relied on 1^st^ (e.g., corn syrup, glucose or starch) and 2^nd^ (e.g., glycerol or xylose) generation feedstocks to synthesize succinic acid (Ahn et al. 2016; Dickson et al. 2021; Li and Mupondwa 2021). The feedstock and its pretreatment are important cost factors for producing bulk chemicals like succinic acid (Straathof 2011; Dickson et al. 2021; Mancini et al. 2022). For instance, independent studies estimated that the expenses for corn syrup contributed 31% to BioAmber's operational cost (Li and Mupondwa 2021), or 67% to the manufacturing costs (Dickson et al. 2021). For 2^nd^ generation substrates like corn stover, the investment and manufacturing costs can further be influenced by more elaborate pretreatment requirements (Dickson et al. 2021). However, the costs of sourcing sustainable next‐generation feedstocks (e.g., formic acid or methanol from CO_2_ hydrogenation powered by wind energy) are often high and exceed those of conventional substrates (Orfali et al. 2025).

Adding to cost concerns and the lacking succinic acid demand, some processes' economic problems were interwoven with sustainability issues and fell short for the impulse that ignited efforts to produce succinic acid. An excessive consumption of acid and base is not only detrimental to the economics, but also to the sustainability of succinic acid biomanufacturing (Dickson et al. 2021; Mancini et al. 2022). Furthermore, utilizing 1^st^ generation substrates for chemical manufacturing competes with food, feed and fibre production and potentially occupies valuable arable land (Jeswani et al. 2020; Bachleitner et al. 2023). For biofuel production, it is established that particularly utilizing 1^st^ generation substrates can lead to losses in biodiversity, water withdrawal, air pollution and environmental acidification and eutrophication (Jeswani et al. 2020). If substrate farming entails a change of land use, such as rainforest deforestation, the greenhouse gas balance of biofuels is often estimated to be worse than that of fossil fuels (Jeswani et al. 2020). Although the production volume of chemicals is much lower than that of fuels (Bachleitner et al. 2023), it still is reasonable to assume that manufacturing bio‐chemicals and biofuels is associated with similar drawbacks. In contrast, valorizing next‐generation feedstocks, such as one‐carbon or plastic fractions, can sequester greenhouse gases or reduce other waste streams and therefore directly contributes to achieve sustainability goals (Stöckl et al. 2022; Tiso et al. 2022; Bachleitner et al. 2023; Wang et al. 2024; Orfali et al. 2025).

Consequently, economic, environmental and social sustainability are interconnected and must have a balanced status for the design of future‐proof succinic acid processes. Thus, advancing succinic acid biosynthesis should synergistically target sustainability and profitability. In this article, we discuss how metabolic engineering of suitable host organisms could contribute to solving sustainability and cost issues of biotechnological succinic acid production in two critical fields: sustainable carbon and energy sources as well as low‐pH fermentations.

The pKa values of the carboxylic acid groups of succinic acid are 4.16 and 5.61 (López‐Garzón and Straathof 2014). Depending on the local pH, succinic acid will therefore exist as a dicarboxylate, monocarboxylate or carboxylic acid (López‐Garzón and Straathof 2014). Thus, we will refer to ‘succinic acid’ in the context of low‐pH fermentations, but to ‘succinate’ in metabolic contexts in the following.

## 2 Metabolic Engineering for Succinic Acid Production From Sustainable Raw Materials

Microbial succinate production from sugars, but also other substrates such as crude glycerol, acetate or carbohydrate‐ and protein‐rich biomass hydrolysates (e.g., from food, agricultural or municipal waste) is well‐established and was reviewed elsewhere (Choi et al. 2015; Ahn et al. 2016; Liu et al. 2022; Mitrea et al. 2024; Schmollack et al. 2024; Korka et al. 2025). To generate possibly far‐reaching sustainability benefits, succinate bioprocesses need to make use of carbon sources which are decoupled from feed and food and have possibly few negative effects on the environment. The greenhouse gas CO_2_ and the CO_2_‐derived organic C1 molecules methane, methanol, formate and CO are considered to be critical substrates for sustainable bioproduction (Tahiraj et al. 2025; Yishai et al. 2016; Cotton et al. 2020; Fackler et al. 2021; Stöckl et al. 2022; Bachleitner et al. 2023). Utilizing plastic waste as substrate for biotechnological succinate manufacturing similarly offers potential to reduce environmental pollution and the global warming impact over fossil fuel‐driven processes (Tiso et al. 2022). However, many metabolic routes to efficiently valorize these carbon streams have remained underexplored so far.

### 2.1 A General Framework for Microbial Succinate Synthesis

**FIGURE 1:**
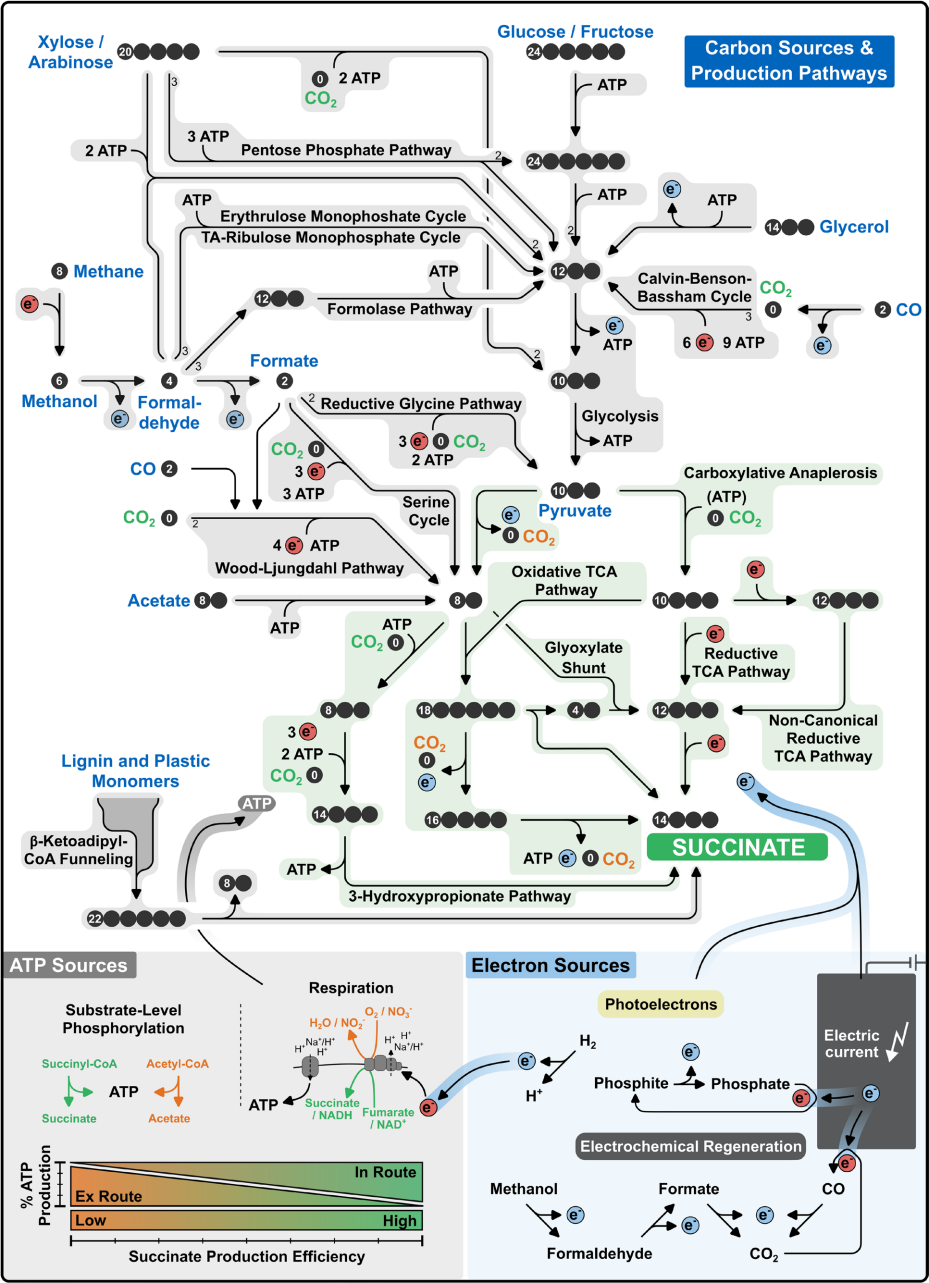
Metabolic pathways for producing succinate from different carbon and energy sources. Black circles represent the number of carbon atoms of each metabolite. The number in the leftmost carbon atom indicates the molecule's degree of reduction. Inorganic carbon species other than CO are indicated as CO_2_, ATP equivalents are indicated as ATP, and hydride transferring redox cofactors and coproducts are indicated as e^−^. Reaction routes for carbon assimilation, succinate biosynthesis, as well as electron and ATP allocation were compiled based on the literature reviewed herein and metabolic pathways collected in the Kyoto Encyclopedia of Genes and Genomes (Kanehisa and Goto 2000).

There is a vast variety of metabolic networks which can potentially realize succinate formation. Different substrates and their metabolization pathways yet have different prerequisites to allow for efficient production. One or more substrates, substrate assimilating routes, succinate synthesis pathways, (reduction) power generating modules and cellular accessories (e.g., transporters and resistance mechanisms) must be considered to design a succinate production scheme (Figure 1). Importantly, their metabolic and biochemical specificities, e.g., redox, carbon and energy balances, thermodynamic driving forces, cofactor preferences or operation conditions (aerobic or anaerobic) must be compatible.

The thermodynamic driving force of newly designed succinate synthesis routes can be estimated using tools like eQuilibrator (Beber et al. 2021) and ultimately defines their feasibility. To assess the biosynthetic capacities of different substrates and biosynthetic schemes, we compiled and visualized their carbon, ATP and redox balances (Figure 1). The redox feasibility and carbon efficiency of a production route are governed by the degree of reduction (*γ*) and carbon content of the substrate(s) and succinate. Glucose, e.g., comprises six carbon atoms and the sugar's *γ* is 24. Succinate features four carbon atoms at a *γ* of 14. Thus, the redox and carbon balances generally only allow to produce one molecule of succinate from one molecule of glucose. If succinate is formed from glucose via the oxidative branch of the tricarboxylic acid cycle (oxTCA), oxidative decarboxylation releases the excess carbon as CO_2_, while the excess electron pairs (five) diverge to other pathways (e.g., respiration) (Figure 1). Still, the product yield can be enhanced by selecting carbon and electron‐preserving routes and providing additional substrates. The glyoxylate shunt of the TCA cycle (gTCA) bypasses the oxidative decarboxylation reactions of the oxTCA and preserves the excess carbon and some electrons as glyoxylate for upcoming synthesis runs. The reductive branch of the TCA cycle (rTCA) bypasses all oxidative decarboxylation steps and fully exploits the biosynthetic potential of its substrates. However, rTCA flux cannot be satisfied with glucose as sole substrate. Instead, two additional carbon entities need to be supplied as CO_2_ (*γ* = 0) and two additional electron pairs (*γ* = 2 per electron pair) need to be available to form two succinate per glucose molecule. Given its carbon and redox efficiency, the rTCA is the preferred pathway for succinate synthesis in the following. Production pathways can also be synergistically interconnected, such that, for example, flux through the oxTCA or gTCA replenishes reduced redox cofactors for the rTCA (Radoš et al. 2014; Vuoristo et al. 2016; Thoma et al. 2022; Cui et al. 2023; Tran et al. 2025). Additional reduction power can also be externally fed to a production host as reduced co‐substrates (e.g., formate (Litsanov et al. 2012)), electron carriers or electric current (Claassens et al. 2018). Vice versa, excess reduction power can be dissipated ex route (e.g., by respiration or by‐product formation) (Weusthuis et al. 2011).

The ATP balance more strongly depends on the thermodynamic landscape and biochemical layout of a metabolic pathway. Vice versa, the energetics of carbon reformation are, e.g., reflected in a pathway's ATP demand or yield, and its trade‐off with the thermodynamic driving force. This applies to glycolytic (Folch et al. 2021) as well as to C1‐fixing pathways (Bar‐Even et al. 2012; Bachleitner et al. 2023; O'Keeffe et al. 2025), and also to succinate synthesis routes (Liu et al. 2022) (Figure 1). If succinate is formed from glucose through the oxidative and decarboxylative oxTCA, 2–3 ATP are yielded by substrate level phosphorylation. Flux through the gTCA which circumnavigates two of the oxidative decarboxylation reactions only produces 1–2 ATP. Carboxylative succinogenesis via the highly carbon and redox efficient rTCA comes at even higher metabolic energy costs and generates 0–2 ATP per metabolized glucose molecule (Figure 1). Notably, this difference in the ATP yield would be even higher, if the associated respirational ATP production would also be considered. The variation in the succinate production schemes' ATP yield above is determined by the involved carboxylative anaplerotic routes.

These reactions feed the TCA variants from the glycolysis end‐products phosphoenolpyruvate or pyruvate in an ATP‐consuming or ATP‐conserving manner (Liu et al. 2022). Carboxylation reactions are thermodynamically challenging (Bar‐Even et al. 2012) and so is the carboxylation of pyruvate to oxaloacetate (∆_*r*_ *G*'^m^ = 39.6 ± 1.4 kJ mol^−1^ (Beber et al. 2021)). Thus, pyruvate carboxylase and phosphoenolpyruvate carboxylase directly or indirectly couple carboxylative oxaloacetate formation to ATP consumption (Liu et al. 2022). Coupling ATP hydrolysis to carboxylation reactions provides a strong thermodynamic driving force (Bar‐Even et al. 2012), and the reactions catalysed by these enzymes favour the carboxylative direction (∆_*r*_ *G*'^m^ = −7.2 ± 1.4 and −32.2 ± 1.6 kJ mol^−1^ (Beber et al. 2021)). Phosphoenolpyruvate carboxykinase catalyses the reversible carboxylation of PEP to oxaloacetate and mechanistically preserves the high energy content of phosphoenolpyruvate by forming ATP (Matte et al. 1997; Carlson and Holyoak 2009). While the phosphoenolpyruvate carboxykinase reaction favours phosphoenolpyruvate formation (∆_*r*_ *G*'^m^ = 14.5 ± 1.6 kJ mol^−1^ (Beber et al. 2021)), the rTCA couples this reaction to the strongly exergonic reduction of oxaloacetate with NADH (∆_*r*_ *G*'^m^ = −26.5 ± 0.6 kJ mol^−1^ (Beber et al. 2021)). Thus, the overall conversion of phosphoenolpyruvate to malate is energetically favourable and channels carbon towards succinate. Consequently, the ATP expenditure of the pyruvate carboxylase and phosphoenolpyruvate carboxylase appears insignificant to ensure flux towards succinate in many production networks. Malic enzyme can catalyse the reductive carboxylation of pyruvate to malate (Stols and Donnelly 1997) and similarly couples the strongly unfavoured carboxylation of pyruvate to the favoured reduction of oxaloacetate. However, this route is energetically unfavoured (∆_*r*_ *G*'^m^ = 13.1 ± 1.5 kJ mol^−1^ (Beber et al. 2021)), which might represent a bottleneck in certain applications.

Various other processes can consume ATP, including cellular maintenance, growth, transport processes and pH homeostasis (see below) (Folch et al. 2021). Import processes are highly substrate specific, but approaches for choosing ATP‐saving transporters are available (Folch et al. 2021). Succinate export was repeatedly engineered to enhance the dicarboxylate's bioproduction, yet detailed information about export mechanisms and energetics is often not available (Beauprez et al. 2011; Fukui et al. 2011, 2019; Chen et al. 2014). Some succinate exporters were characterized more in detail, e.g., fumarate, l‐tartrate and citrate antiporters, as well as a voltage‐dependent slow‐anion channel transporter (Janausch et al. 2002; Kim and Unden 2007; Darbani et al. 2019). Given their impracticability as (co‐)substrates for industrial succinate production, we expect the dicarboxylate/succinate antiporters to be of ancillary importance. However, antiporters that couple the import of relevant substrates with succinate export could be of interest for efficient succinate biosynthesis. In *Escherichia coli* , succinate export during glucose fermentation is thought to be mediated by H^+^/succinate symport (Janausch et al. 2002). Similarly, succinate extrusion was estimated to be associated with the export of 2.3–2.7 protons in *Corynebacterium glutamicum* , while the mechanism of transport remained unclear (Radoš et al. 2014). Notably, such secondary H^+^/succinate symport contributes to building the proton motive force and, consequently, can generate metabolic energy (Konings et al. 1995). However, this type of transport and energy conservation is expected to be thermodynamically unfavourable at high extracellular product concentrations and low pH (van Maris et al. 2004). Consequently, active carboxylate extrusion is desirable in industrial applications albeit being energy intense (van Maris et al. 2004). An energy‐efficient solution for succinate export at low pH in yeast is Mae(p) from *Schizosaccharomyces pombe* (Darbani et al. 2019). SpMae(p) is assumed to couple succinate export to the voltage gradient rather than ATP expenditure or ion transport (Darbani et al. 2019).

High succinate yields are achievable if required ATP is generated in route, i.e., in reactions immediately involved in substrate assimilation and succinate synthesis (Folch et al. 2021). Besides substrate level phosphorylation, in route ATP production can also proceed through respiration (Figure 1) (Folch et al. 2021). Fumarate respiration couples electron transport phosphorylation to exergonic reductive succinic acid formation (Kröger et al. 2002; Folch et al. 2021). Moreover, acetogens harness electron bifurcation to build an ion motive force while preserving reduced redox cofactors for C1 assimilation (Schuchmann and Müller 2014; Folch et al. 2021). Without sufficient in route ATP production, some fraction of the carbon and/or reduction power needs to be sacrificed to drive ATP regeneration in pathways outside of succinic acid synthesis (ex route) (Weusthuis et al. 2011, 2020; Folch et al. 2021). Ex route ATP synthesis can likewise proceed via respiration or substrate‐level phosphorylation (Weusthuis et al. 2011, 2020, Folch et al. 2021). However, ex route ATP regeneration will reduce the efficiency of succinic acid biosynthesis and, therefore, must be cautiously controlled (Figure 1) (Weusthuis et al. 2011, 2020, Folch et al. 2021). In the following, we will discuss ATP, redox and carbon balances as well as relevant thermodynamic aspects to retrace strategies for resource‐efficient succinate biosynthesis from next‐generation substrates.

### 2.2 Waste Fractions

Succinate production from various carbohydrate‐, protein‐ and glycerol‐rich waste‐ and side‐products was repeatedly demonstrated (Mitrea et al. 2024). In contrast, exploiting recalcitrant lignin and plastic waste for succinate biosynthesis remains poorly explored. Both carbon pools are increasingly being considered as substrates for biotechnological processes, and their metabolization routes are partially cognate (Becker and Wittmann 2019; Ru et al. 2020; Tiso et al. 2022). In plastics and lignin metabolism, a broad spectrum of raw materials is channelled into few common intermediates which can build up a variety of products (Becker and Wittmann 2019; Tiso et al. 2022). Exemplarily, depolymerization and degradation of different lignins and plastics, including poly(ethylene terephthalate), polystyrene, polyamine and polyol polyurethane can converge in β‐ketoadipate derivatives (Tischler 2015; Becker and Wittmann 2019; Ru et al. 2020; Tiso et al. 2022). Their degradation eventually generates acetyl‐ and succinyl‐CoA (Tischler 2015, Becker and Wittmann 2019, Ru et al. 2020, Tiso et al. 2022) and feeds the central metabolism in the immediate vicinity of succinate (Figure 1). This funnelling mechanism might enable developing parallel strategies to transform (mixed) lignins and plastics into succinate. Still, the structural wealth of lignins, plastics and their degradation pathways could allow for numerous other efficient succinate synthesis setups (Becker and Wittmann 2019, Ru et al. 2020, Tiso et al. 2022). The decomposition of synthetic lignin into succinate was demonstrated with a basidiomycete but is impeded by a very low performance (Table 1) (Hong et al. 2017). Forming succinate from plastic waste was recently proposed (Ru et al. 2020, Tiso et al. 2022), but we are not aware of a practical implementation of such a synthesis route yet.

### 2.3 One‐Carbon Substrates

The simplest approach to utilize C1 substrates for succinate production is to primarily extract their reduction power for boosting the conversion of sugars (Litsanov et al. 2012; Ahn et al. 2017; Guo et al. 2022; Tao et al. 2025). Concomitantly, the final oxidation product of C1 molecules, CO_2_, can be rerouted into succinate via carboxylative anaplerosis (Litsanov et al. 2012, Ahn et al. 2017, Guo et al. 2022, Tao et al. 2025). However, the assimilation of formate‐derived CO_2_ is inefficient. Less than 20% of the succinate produced with *Mannheimia succiniciproducens* from glucose and formate contained formate‐derived carbon (Ahn et al. 2017). Alternative native and non‐native assimilation routes can intensify the conversion of C1 substrates into succinate (Figure 1). Many C1 metabolization pathways are interwoven and comprise common intermediates and reaction routes (Tahiraj et al. 2025; Cotton et al. 2020; O'Keeffe et al. 2025). Highly reduced methane (*γ* = 8) and methanol (*γ* = 6) are mostly oxidized to yield formaldehyde (*γ* = 4) or formate (*γ* = 2), which can serve as building blocks for more complex metabolites through various pathways (Tahiraj et al. 2025, Cotton et al. 2020, O'Keeffe et al. 2025).

Methane features the highest *γ* = 8 of all C1 molecules, and two methane molecules could satisfy the redox demand of succinate synthesis from a balancing perspective. However, microbially accessing methane is energetically challenging (Dinh and Allen 2024). Also, aerobic methane oxidation cannot make use of the substrate's high *γ*, as it consumes an electron pair and molecular oxygen (Tahiraj et al. 2025). Thus, at least four methane molecules are needed to form one succinate molecule aerobically. In contrast, the oxygen‐susceptible methyl coenzyme M reductases can shuffle the electrons from methane oxidation to a redox cofactor (Dinh and Allen 2024). This reaction, however, is thermodynamically unfavourable, and methyl coenzyme M reductases are highly complex. Thus, applying methyl coenzyme M reductase in biotechnological methane valorization is expected to be limited at short notice (Dinh and Allen 2024).

Methanol (*γ* = 6) is more accessible, and three molecules of methanol can be enough to fulfil succinogenic redox demand while providing surplus redox power (two electron pairs) which may drive ex route processes. Thus, methanol is the only C1 substrate which is sufficiently reduced to enable additional CO_2_ fixation (*γ* = 0) as co‐substrate (i.e., not originating from in route C1 oxidation). However, the potential of oxidizing methanol to formaldehyde is only moderately negative (*E*'^m^ ~ −185 mV (Beber et al. 2021)). This represents a thermodynamic obstacle for coupling methanol oxidation to the reduction of NAD^+^ (Claassens et al. 2018). Thus, noncanonical redox cofactors of tweaked reduction potential are discussed to extract methanol's reduction power more efficiently for reductive biosynthesis (Orsi et al. 2024). Notably, the potential of fumarate reduction is relatively high (*E*'^m^ ~ −42 mV (Beber et al. 2021)) and might be couplable to methanol oxidation via noncanonical redox cofactors.

Formaldehyde represents a versatile functional building block in C1 metabolism, and four formaldehyde moieties (*γ* = 4) feature sufficient redox capacity to theoretically allow for succinate synthesis. Formaldehyde assimilating routes, such as the ribulose monophosphate (RuMP) cycle, the xylulose monophosphate (XuMP) cycle or the synthetic erythrulose monophosphate (EuMP) cycle and formolase pathway often build the glycolysis intermediate glyceraldehyde‐3‐phosphate (Figure 1) (Anthony 1982; Siegel et al. 2015; Cotton et al. 2020; Wu et al. 2023). The RuMP and XuMP cycle, as well as their variants, differ in ATP demand and thermodynamic driving force (Cotton et al. 2020; Bachleitner et al. 2023). The most ATP efficient RuMP/XuMP cycle variant is the transaldolase (TA)‐RuMP cycle. While the chemical driving force of this pathway is rather low, it requires only one ATP to form glyceraldehyde‐3‐phosphate from formaldehyde and yields one ATP if glyceraldehyde‐3‐phosphate is metabolized to pyruvate via glycolysis (Bachleitner et al. 2023). Making use of this ATP‐yielding formaldehyde assimilation route, the methanotroph *Methylomicrobium alcaliphilum* 20Z imitates fermentation under oxygen‐limited conditions to produce reduced end‐products including succinate from methane (Kalyuzhnaya et al. 2013). An oxygen‐dependent switch to fermentation‐like behaviour was recently also found to boost succinate production from methane in a tailor‐made *Methylotuvimicrobium buryatense* 5GB1S strain (Wang et al. 2024) and was potentially also observed for an engineered *Methylomonas* sp. DH‐1 variant (Nguyen et al. 2019). The formaldehyde fixing RuMP module was recently also introduced to a tailored *E. coli* strain to enhance fermentative succinate production from glucose with methanol as additional carbon and electron source, yet the benefit was restricted (Zhang et al. 2018). In a similar approach, the XuMP cycle was functionally established in *Yarrowia lipolytica* to produce 0.92 g succinic acid L^−1^ using methanol as main carbon source (Table 1) (Zhang et al. 2023). Being independent of any formaldehyde acceptor, the synthetic formolase allows to sequentially ligate formaldehyde entities to yield dihydroxyacetone in an exergonic reaction sequence (Siegel et al. 2015). Like the TA‐RuMP cycle, the formolase pathway or the EuMP cycle require only one ATP to assimilate formaldehyde into glyceraldehyde‐3‐phosphate (Siegel et al. 2015; Wu et al. 2023) and, thus, yield ATP upon glycolytic pyruvate formation (Figure 1). These ATP‐yielding formaldehyde assimilation routes can be coupled to the rTCA to yield ATP‐neutral or ATP‐positive schemes for succinate production. Still, some substrate capacities likely need to be catabolized ex route to power homeostasis or transport‐related energy consumption. Assimilating formaldehyde with pentose derivatives (Anthony 1982), modules of the RuMP and XuMP cycles can also be employed for co‐utilizing C1 substrates and pentoses such as xylose (Zhang et al. 2023). The combined *γ* of xylose and formaldehyde is the same as that of glucose (*γ* = 24). Also, the ATP balance of co‐utilizing xylose and formaldehyde resembles that of glucose (Figure 1).

Further oxidation of formaldehyde produces formate (*γ* = 2) of which at least seven molecules are required to satisfy the redox demand of succinate formation. Thus, formate represents the only organic C1 compound of which four molecules cannot build up the C4 molecule succinate due to redox constraints. Thus, employing formate as substrate for succinate synthesis is associated with liberating CO_2_, even if the formate oxidation needed to power the assimilation pathways' ATP demand is neglected. There are several options for formate assimilation, including the reductive glycine pathway, the serine cycle or the Wood‐Ljungdahl pathway (Yishai et al. 2016; Cotton et al. 2020). These pathways often also fix CO_2_, differ in their ATP and redox demand, and yield different products (Yishai et al. 2016). Many of these pathways, however, need to be driven by ATP hydrolysis (Yishai et al. 2016), implying that further formate (or other co‐substrate) metabolization is likely required for ex route ATP regeneration. The serine cycle was, e.g., likely also involved in the production of succinate by aforementioned methanogens (Nguyen et al. 2019; Jo et al. 2024; Wang et al. 2024). Notably, the serine cycle can yield succinate from two formate and two CO_2_ in a concerted action with the gTCA reactions, consuming five ATP and five electron pairs (Anthony 1982; Bar‐Even et al. 2013). Still, the serine cycle is rather ATP intense and structurally similar to glycolysis and the TCA cycle, which might cause the orchestration of metabolic fluxes to be quite complex (Yishai et al. 2016). The reductive glycine pathway is more ATP efficient at a reasonable chemical driving force and accumulates the C3 body pyruvate (Figure 1) (Bar‐Even et al. 2013). Consequently, the reductive glycine pathway can support succinogenesis via the rTCA, yet this network relies on ex route ATP regeneration. While the reductive glycine pathway was used to, e.g., produce lactate (Kim et al. 2023), we are unaware of its application in succinate bioproduction. Li et al. (2025) demonstrated using formate as sole carbon and energy source for succinate production with a recombinant *Vibrio natriegens* strain (Table 1). To establish the assimilation of formate and CO_2_ in *V. natriegens* , the tetrahydrofolate cycle was introduced. Remarkably, the used formic acid was produced by electrochemically capturing and reducing oceanic CO_2_ (Li et al. 2025).

The final oxidation product of C1 molecules is CO_2_. While CO_2_ sourcing offers economic and environmental advantages over many other carbon sources (Orfali et al. 2025), its biotechnological valorization is challenging. Accordingly, few studies reported direct succinate production from CO_2_ as sole carbon source. As CO_2_ is completely oxidized (*γ* = 0), any succinate (*γ* = 14) production route involving CO_2_ is inherently electron intense (Figure 1). Moreover, CO_2_ fixation is thermodynamically unfavoured and, thus, must be coupled to strongly exergonic reactions, such as ATP hydrolysis (Bar‐Even et al. 2012). There are a variety of CO_2_‐fixing pathways (e.g., the 3‐hydroxypropionate cycle, Wood‐Ljungdahl pathway, Calvin‐Benson‐Bassham cycle (CBB) or the reductive tricarboxylic acid cycle) which follow different energetic profiles (Bar‐Even et al. 2012; Bachleitner et al. 2023). The CBB cycle is the dominant route for carbon fixation in nature (Berg 2011) and offers a reasonable chemical driving force (Bar‐Even et al. 2013; Bachleitner et al. 2023). Concomitantly, CO_2_ fixation via the CBB cycle is highly ATP‐intensive (Bar‐Even et al. 2012). Thus, any succinate production route based on CBB cycle‐mediated CO_2_ assimilation is ATP‐negative and must be coupled to ex route ATP production (Figure 1). The succinate biosynthesis pathway to be coupled with the CBB cycle appears to be freely selectable, as long as sufficient redox power is available. Lai et al. (2022) used the cyanobacterium *Synechococcus elongatus* PCC 7942 to produce 8.9 g succinic acid L^−1^ via a variant of the oxTCA from atmospheric CO_2_ and photoenergy (Table 1). To our knowledge, there are no reports about systematically tailoring aerobic or anaerobic chemolithoautotrophic organisms for succinate production.

Making use of such organisms, gas fermentation is an industrially applied process for the conversion of CO_2_ into bulk chemicals (Fackler et al. 2021). Gas fermentations can rely on the anoxic assimilation of CO_2_ or CO into acetyl‐CoA via the Wood‐Ljungdahl pathway, which is coupled to the oxidation of CO or H_2_ and consumes one ATP (Fackler et al. 2021). Succinate was identified as overflow metabolite in *Clostridium autoethanogenum*, showcasing that the production of the dicarboxylate from CO_2_ via the Wood‐Ljungdahl pathway might be feasible (Marcellin et al. 2016). Since an industrially relevant accumulation of succinate based on the Wood‐Ljungdahl pathway is yet to be demonstrated, the metabolic scheme to do so remains elusive. Like acetogenesis, the biosynthesis pathway should preferably be coupled to in route ATP production (e.g., via the succinyl‐CoA node), to avoid forfeiting biosynthetic capacities in ex route ATP generation (e.g., via acetate co‐production) (Figure 1). Alternatively, Liebal et al. (2018) evaluated a biosynthesis scheme in which acetate is the product of anaerobic gas fermentation which is then aerobically used by a second strain to yield succinate.

The CO_2_‐fixing 3‐hydroxypropionate cycle and 3‐hydroxypropionate‐4‐hydroxybutyrate cycle share several reactions (Berg et al. 2007) which provide an additional route for succinate biosynthesis (Liu et al. 2020; Li et al. 2024). This pathway branches from the central carbon metabolism at acetyl‐CoA. If acetyl‐CoA is formed by non‐decarboxylative means, e.g., by fatty acid catabolism, this route allows fixing two CO_2_ molecules per formed succinate at the cost of two ATP (Li et al. 2024). If, however, acetyl‐CoA is produced from pyruvate decarboxylation, this pathway fixes as much CO_2_ per succinate molecule as the rTCA but consumes more ATP (Figure 1).

Like formaldehyde assimilation, CO_2_ fixation can be tied to sugar metabolization. For this reason, two key enzymes of the CBB cycle, the ribulose bisphosphate carboxylase and the phosphoribulokinase, were repurposed for producing succinate from xylose and CO_2_ via the rTCA under anaerobic conditions (Zhou et al. 2025). Yet, this approach suffered from low efficiency (Table 1). The combined *γ* of xylose and CO_2_ is 20, indicating that the reductive capacity of this substrate combination only suffices to produce one molecule of succinate per xylose and CO_2_. Consequently, succinate yield can only be optimized if four further electron pairs are provided. Moreover, coupling the co‐assimilation of CO_2_ and xylose via the PRK‐RuBisCo pathway to a fully capacitating rTCA is ATP‐neutral, or ATP‐negative (Figure 1). Concomitantly, Zhou et al. (2025) found the succinate yield in this strain background to strongly depend on ATP conservation during the formation of oxaloacetate and malate. Thus, efficiently producing succinate from CO_2_ and xylose requires a high supply of electrons, which drive not only reductive product formation but potentially also ATP regeneration.

### 2.4 Electron Sources and Electronic Circuits

In many cases, efficiently producing succinate is highly electron‐intensive (Figure 1). Approaches to feed producers with electron‐donating co‐substrates were repeatedly explored, specifically using formate, but also methanol (Litsanov et al. 2012; Ahn et al. 2017; Guo et al. 2022; Tao et al. 2025). However, the relatively high reduction potential of methanol oxidation might impede its use as electron donor (see above). In addition, there are several other attractive electron donors, such as H_2_ or CO, which can efficiently be regenerated electrochemically to deliver reduction power into a cell (Figure 1) (Claassens et al. 2018). Among them is also the non‐toxic and well‐soluble phosphite (Claassens et al. 2018). The oxidation of phosphite to phosphate is characterized by a very low reduction potential, but its electrochemical regeneration yet is inefficient (Claassens et al. 2018). The electrochemical regeneration of electron donors can be combined with or separated from their biotechnological utilization (Claassens et al. 2018; Stöckl et al. 2022). In combined approaches, natural red or H_2_ can, for example, shuttle electrons between an electrode and succinate producing cells (Pateraki et al. 2019; Wu et al. 2019). Electrons can also be directly conducted between cells and electrodes, but low current densities might limit these approaches' production performance (Claassens et al. 2018). Allocating redox cofactors in (semi‐)synthetic metabolic networks designed to efficiently produce a specific product is highly non‐trivial. If some fraction of a substrate's redox power is required to drive ATP generation, controlling the electron flux in cells can quickly pose a serious hurdle for efficient biosynthesis (Weusthuis et al. 2020). Employing non‐canonical redox cofactors in tailor‐made electronic metabolic circuits can help to harmonize redox allocation (Weusthuis et al. 2020), and is expected to be specifically important for C1‐based bioproduction (Orsi et al. 2024).

## 3 Metabolic Engineering for Low‐pH Succinic Acid Fermentations

To supersede the need for pH titration and to reduce the overall salt load during succinic acid production, natively or synthetically acid‐tolerant or acidophilic production hosts are crucial (see above). To date, yeasts' acid tolerance is a competitive edge over bacteria in the production of succinic acid at low‐pH conditions (Zhong et al. 2024; Korka et al. 2025) (Table 1). However, yeasts' compartmentalization can also complicate metabolic engineering (Cui et al. 2023; Tran et al. 2025). Indeed, early‐stage succinic acid production with yeast suffered from low performance (compare key performance indicators listed by, e.g., Korka et al. (2025) and Schmollack et al. (2024)). The yield, however, is a critical factor for the cost‐effectiveness of low‐pH succinic acid production processes (Tran et al. 2023, 2025). Recent years brought considerable advancements in the development of low‐pH production processes for succinic acid with acid‐tolerant yeasts, specifically with *Yarrowia lipolytica* and *Issatchenkia orientalis* (Cui et al. 2023; Tran et al. 2023, 2025; Zhong et al. 2024; Tao et al. 2025, 2026).

One key to this development was the implementation of the rTCA to enhance the efficiency of succinic acid production. Critically, the cytosolic expression of the rTCA did not suffice for highly efficient production, as it did not allow for redox balancing through mitochondrial oxidative succinic acid pathways. Targeting rTCA enzymes to the mitochondrial matrix or reversely localizing copies of the pyruvate dehydrogenase and the glyoxylate pathway to the cytosol eventually allowed for high succinic acid yields from glucose in independent approaches (Cui et al. 2023, Tran et al. 2025). Scaled processes using these strains enabled efficient succinic acid production from glucose at pH 3 with *I. orientalis* (Tran et al. 2025), or without pH control with *Y. lipolytica* (Cui et al. 2023) (Table 1). The cytosolic redox imbalance was later also addressed by changing the cofactor dependency of reductive succinic acid formation by using a non‐canonical rTCA (Tao et al. 2026). To date, the highest glucose‐specific succinic acid yield in yeast (1.01 g g^−1^) was achieved using this strategy in a *Y. lipolytica* strain which also overexpressed a carbonic anhydrase to support CO_2_ assimilation (Table 1) (Tao et al. 2025). The pH was not controlled during this process and was lower than 3 by the end of the fermentation (Tao et al. 2025).

Albeit yeasts feature a high natural tolerance against acidic stressors, their robustness can further be enhanced. For instance, a lactic acid‐producing *Saccharomyces cerevisiae* strain was subjected to increasing product concentrations at decreasing pH values, which eventually enhanced its low‐pH fermentation performance by up to 250% (Altvater et al. 2025). Also, yeast‐mediated succinic acid synthesis only now starts to approach the performance that can be achieved with bacterial producers (Table 1). Exemplarily, the succinic acid productivity of a recombinant *V. natriegens* strain was found to be as high as 8.5 g succinate L^−1^ h^−1^ (Schulze et al. 2024), which still is out of yeast systems' reach (Table 1). Moreover, a succinic acid production process with *C. glutamicum* could be operated for 572 h with cell recycling and yielded 634 g succinic acid (Jojima et al. 2016). Consequently, using acid‐tolerant succinic acid producing bacteria could further improve low‐pH process performances. While *E. coli* is rather acid‐tolerant (Kanjee and Houry 2013), existing prokaryotic succinic acid processes have been operated at near‐neutral pH. Also, there barely are domesticated prokaryotes which withstand highly protic environments in white biotechnology. Thus, novel hosts for succinic acid production should be acid‐tolerant, acidophilic or tolerate a broad pH range. Several acidophilic organisms, which can grow at pH values of 3 and lower, are increasingly considered potential biotechnological chassis, but their product spectrum and applicability are still rather restricted and poorly explored (González et al. 2024). Nonetheless, to suitably equip a succinic acid producer for acidic environments in the context of highly efficient, artificial production networks, it is important to understand the main principles of acid tolerance. To this end, selected acid tolerance mechanisms and their applicability for succinic acid biosynthesis will be briefly discussed in the following:

To elude from acid stress, cells possess some main tactics which include blocking proton intrusion, proton sequestration, intracellular pH buffering and managing cellular macromolecules (Baker‐Austin and Dopson 2007; Kanjee and Houry 2013; Shabayek and Spellerberg 2017; Guan and Liu 2020).

Proton sequestration: pumping—An important cellular mechanism for coping with acid stress is proton extrusion through proton‐pumping hydrogenases and ATPases (Kanjee and Houry 2013; Guan and Liu 2020). *E. coli* is thought to employ a variety of dehydrogenases to increase proton extrusion in moderately acidic environments (Kanjee and Houry 2013). F‐type ATPases were reported to be involved in acid resistance in a broad variety of microorganisms (Guan and Liu 2020). Eukaryotes further possess V‐type and P‐type ATPases for pH homeostasis (Folch et al. 2021). These proton‐translocating proteins are, however, closely chained to the cellular energy and redox metabolism (Kanjee and Houry 2013; Guan and Liu 2020; Folch et al. 2021) and their involvement in acid resistance will impede the possible succinic acid yield.

Proton sequestration: buffering—Protons can also be eliminated in intracellular reactions. The formate hydrogen lyase complex, for instance, reduces protons to H_2_ by the oxidation of formate in acid‐stressed *E. coli* (Kanjee and Houry 2013). While formate may serve as substrate for succinic acid synthesis anyway (Figure 1), this mechanism withdraws formate from biosynthesis. Moreover, sequestering protons as H_2_ is infeasible if H_2_ is used as an electron donor. Alternatively, protons can be consumed in ammonia‐ or CO_2_‐generating reactions (Kanjee and Houry 2013, Shabayek and Spellerberg 2017, Guan and Liu 2020). The involved amino acid decarboxylases and deaminases are coupled to substrate/product antiporters to enable sustained proton sequestration (Kanjee and Houry 2013). Nonetheless, externally supplying amino acids for pH homeostasis only shifts pH control and the accompanying drawbacks from the fermentation broth to the intracellular space.

Macromolecule management—There are various mechanisms dedicated to the management of cellular macromolecules during acid stress (Cotter and Hill 2003; Kanjee and Houry 2013; Guan and Liu 2020). A bouquet of chaperons including the periplasmic HdeA and HdeB, the cytosolic Hsp31 and the cytosolic ATP‐dependent GroEL/ES, DnaK, DnaJ, or GrpE can be involved in protein folding, shielding, renaturation or degradation (Kanjee and Houry 2013; Shabayek and Spellerberg 2017). Also, an enhanced proteolytic degradation of acid‐denatured proteins was hypothesized to benefit succinic acid tolerance of an engineered *Y. lipolytica* strain (Zhong et al. 2024). At a pH of 3.5, this strain produced 112.54 g succinic acid L^−1^ from glucose which ranges among the highest succinic acid titres in low‐pH fermentations to date (Table 1) (Zhong et al. 2024). DNA management systems such as Rec, Uvr or Smn are dedicated to repair damaged DNA during acid stress (Cotter and Hill 2003; Guan and Liu 2020). While efficiently managing DNA and proteins is an inherently important aspect of any host's acid resistance, we expect macromolecule management as primary acid resistance mechanism to be inefficient.

Blocking proton intrusion—Microorganisms can brace for acid stress by building an acid‐ and proton‐impermeable cell envelope (Baker‐Austin and Dopson 2007; Kanjee and Houry 2013; Guan and Liu 2020; Li et al. 2023). Modifications of the cell envelope may be synthetically introduced to enhance a host's acid tolerance (Guan and Liu 2020, Li et al. 2023). Among the cell envelope modifications which are relevant for proton and acid resistance are, e.g., the content of unsaturated, trans‐unsaturated and cyclopropane fatty acids in bacteria (Chang and Cronan 1999; Ahn et al. 2018; Xu et al. 2020), of tetraether lipids in archaeal membranes (Baker‐Austin and Dopson 2007) or of ergosterol and sphingolipids in yeast (Li et al. 2023). In *E. coli* , an increase in membranous unsaturated fatty acids did not only restore growth at pH 4.2, but also superseded the need for pH control during 3‐hydroxypropionate production (Xu et al. 2020). Designing a robust and impermeable cell envelope appears to be the preferable option to protect succinic acid‐producing microorganisms from acid stress. This mechanism circumvents many disadvantages (active energy consumption, media supplements) of other acid tolerance mechanisms (see above). However, engineering cell envelopes is not yet well established, and genetic modifications that allow the construction of an acid‐resistant cell envelope but do not result in unforeseen side effects need to be uncovered.

Notably, the demand for base (and acid) during succinic acid production at circumneutral pH can also be decreased by in situ product removal. Pateraki et al. (2019) and Stylianou et al. (2023) successfully used electrochemical membrane extraction to remove succinic acid in situ. The concomitant electrochemical production of hydroxide ions reduced the NaOH requirements for pH control by more than 30%, allowing (renewable) electricity to partly take over pH control (Pateraki et al. 2019; Stylianou et al. 2023). Notably, electrochemical membrane extraction also inheres potential to provide cellular reduction power in situ, e.g., by electrolytic H_2_ production (Pateraki et al. 2019). However, this technology can also remove inorganic ions such as bicarbonate or phosphate (Pateraki et al. 2019) which might interfere with certain biosynthesis schemes (Figure 1). Further possibilities for in situ succinic acid removal were reviewed by López‐Garzón and Straathof (2014).

## 4 Outlook and Political Drivers

The applicability of biotechnological succinic acid production has been considerably enhanced in recent years. Specifically, low‐pH production performances—which are crucial for the competitiveness and sustainability of succinic acid production—have improved significantly. In contrast, utilizing low‐impact, next‐generation substrates for succinic acid biosynthesis is limited by these feedstocks' costs and poor microbial production performances. In this opinion, we outlined and discussed strategies for upgrading a variety of next‐generation substrates into succinic acid and argue for rationally adapting well‐defined acid resistance mechanisms. However, such developments are time‐consuming and innovative succinic acid production systems currently suffer from low performance. Consequently, some types of economic sustainability acknowledgement are important tools to reward building lowest‐impact succinic acid production processes over time and foster bio‐succinic acid market penetration. Carbon credits can clearly reduce the price of biotechnological succinic acid (Liang et al. 2023) and might prove critical for successful commercialization, alongside continuous reductions in process costs.

## Author Contributions

**Bastian Blombach:** conceptualization, funding acquisition, writing – original draft, writing – review and editing. **Christoph Gunkel:** conceptualization, writing – original draft, writing – review and editing.

## Funding

This work was supported by the German Federal Ministry for Transport, 16RK34003H.

## Conflicts of Interest

The authors declare no conflicts of interest.

## Data Availability

The authors have nothing to report.
